# The Effects of Obesity on the Inflammatory, Cardiovascular, and Neurobiological Responses to Exercise in Older Adults

**DOI:** 10.3390/biology12060865

**Published:** 2023-06-15

**Authors:** Brandon G. Fico, Arun Maharaj, Gabriel S. Pena, Chun-Jung Huang

**Affiliations:** 1 Department of Kinesiology, University of Wisconsin-Madison, Madison, WI 53706, USA; bfico@wisc.edu; 2 Department of Epidemiology and Cancer Control, St. Jude Children’s Research Hospital, Memphis, TN 38105, USA; arun.maharaj@stjude.org; 3 Department of Kinesiology, University of Maryland, College Park, MD 20742, USA; gpena1@terpmail.umd.edu; 4 Exercise Biochemistry Laboratory, Department of Exercise Science and Health Promotion, Florida Atlantic University, Boca Raton, FL 33431, USA

**Keywords:** obesity, aging, inflammation, vascular function, neurobiology, aerobic exercise, resistance exercise

## Abstract

**Simple Summary:**

Our growing aging population and increased prevalence of obesity have become an emerging health problem as these conditions lead to the development of related diseases. Specifically, obesity and aging are associated with cardiovascular disease and a decline in cognitive function underlying the inflammatory mechanisms. However, the literature regarding how both interact and impact these complex physiological processes across the lifespan remains to be elucidated. As such, this review discusses how obesity in aging adults mediates inflammatory, cardiovascular, and neurobiological effects of exercise in this population.

**Abstract:**

Obesity with advancing age leads to increased health complications that are involved in various complex physiological processes. For example, inflammation is a critical cardiovascular disease risk factor that plays a role in the stages of atherosclerosis in both aging and obesity. Obesity can also induce profound changes to the neural circuitry that regulates food intake and energy homeostasis with advancing age. Here we discuss how obesity in older adults impacts inflammatory, cardiovascular, and neurobiological functions with an emphasis on how exercise mediates each topic. Although obesity is a reversible disorder through lifestyle changes, it is important to note that early interventions are crucial to prevent pathological changes seen in the aging obese population. Lifestyle modifications such as physical activity (including aerobic and resistance training) should be considered as a main intervention to minimize the synergistic effect of obesity on age-related conditions, such as cerebrovascular disease.

## 1. Introduction

Biological aging is associated with increased visceral adipose tissue [1] and a shift in energy homeostasis that often leads to sarcopenic obesity [2] and subclinical chronic pro-inflammation (e.g., inflammaging) [1]. These chronic obese and age-related inflammation are precursors to severe health conditions such as hypertension, dyslipidemia, type 2 diabetes, stroke, coronary artery disease, and various forms of cancer [3]. Many of these complications manifest with normal aging but are more prevalent with age-related obesity as more than 65% of adults in the United States are overweight or obese, substantially increasing the risk of morbidity and mortality [4]. With relative risk of mortality increasing between 27–93% depending on severity of obesity [5]. Particularly, inflammation is a critical cardiovascular disease risk factor that plays a role in the process of atherosclerosis in both aging and obesity. This chronic inflammatory disease is involved with endothelial cell permeability, accumulation of low-density lipoproteins as a major source of atherosclerotic lipid storage, and leucocyte trafficking, eventually leading to endothelial/vascular dysfunction [6]. Additionally, the literature has demonstrated that obesity can induce profound changes to the neural circuitry that regulates food intake and energy homeostasis [7], thereby affecting cognitive development [8]. In this regard, research has previously found a negative relationship between body mass index (BMI) and cognitive test performance, even after controlling for age [9]. Lifestyle modifications including diet and exercise are proposed as a first-line defense to counteract obesity. Thus, while exercise has been extensively shown to effectively provide many physiological health benefits relative to other therapeutic treatments, this review provides an overview of how aerobic exercise, resistance exercise, or in combination would modulate inflammatory, cardiovascular, and neurobiological responses in older adults with obesity based on the latest available data. We anticipated that exercise interventions would prove effective at improving inflammatory, cardiovascular, and neurobiological responses in older adults with obesity. 

## 2. The Effect of Exercise on Inflammation in Obese Older Adults

Obese individuals have immune dysfunction leading to higher infection rates and impaired wound healing [10]. Excess body fat increases leukocyte count (neutrophils, monocytes) but lowers T- and B-cell mitogen-induced proliferation [10]. Obesity-related inflammation is mediated by excessive adipose tissue storage and subsequent apoptotic-related macrophage infiltration, as well as neutrophil, CD4^+^ and CD8^+^ T cell recruitment that consequently leads to insulin resistance within the adipose tissue [11,12]. With macrophage infiltration and the associated metabolic shift from glycolytic to oxidative, reactive oxygen species (ROS) are produced due to the electron transport chain and respiration being attenuated, which allows for the upshot in ROS production, signaling the production of pro-inflammatory cytokines [13]. This overproduction of ROS has been linked to oxidative stress and inflammation [14]. Pro-inflammatory cytokines that are associated with obesity and aging include tumor necrosis factor α (TNF-α), interleukin 6 (IL-6), and C-reactive protein (CRP), which cause peripheral blood mononuclear cells to be in a pro-inflammatory state [15]. This pro-inflammatory profile changes the macrophage polarization state from the M2 (anti-inflammatory) to the M1 (pro-inflammatory) phenotype [16]. Additionally, the ratio of CD8^+^ to CD4^+^ T-cells have been shown to increase with obesity, limiting the secretion of anti-inflammatory cytokines that inhibit macrophage migration from CD4^+^ regulatory T-cells [17,18]. In fact, obesity can lead to accelerated aging, and older adults that are obese have impaired immune responses, for example in response to vaccination [19]. Obesity and age-related increases in inflammatory cytokines (e.g. TNF-α, IL-6, and CRP) cause exacerbated danger-associated molecular patterns and immunosenescence, thereby increasing morbidity and mortality [20].

Adipocytes release signaling molecules (adipokines), including leptin and adiponectin, that have immunomodulatory actions [21]. While leptin has effects on the central nervous system to stimulate satiety and energy expenditure [22], an increase in the circulating levels with obesity can lead to leptin resistance, resulting in the activation of immune cells [23]. Specifically, leptin stimulates monocyte proliferation and differentiation into macrophages, modulating the activation of natural killer cells (NK cells) and stimulating the release of pro-inflammatory cytokines such as TNF-α, IL-6, and IL-12 [10,24]. In contrast, adiponectin is an anti-inflammatory and insulin-sensitizing hormone that is inversely related to body weight and has opposite immunomodulatory actions from that of leptin [25]. In macrophages, adiponectin inhibits phagocytosis and decreases the production of TNF-α [23]. Adiponectin prevents the differentiation of monocytes and lowers the production of endothelial cell adhesion molecules [26]. Adiponectin also induces the production of the anti-inflammatory cytokines IL-10 and IL-1 [24]. 

Importantly, exercise is a main lifestyle modification that is considered a first-line defense to combat age-related obesity by counteracting positive energy balance and also modulate immune cells, adipokines, and inflammatory cytokines [27]. Our previous work showed that young adults with obesity exhibited a comparable concentrations of IL-6 to acute aerobic exercise when compared to normal-weight counterparts [28]. Additionally, these young adults with obesity elicited greater microtubule-associated protein 1A/1B-light chain 2 (LC3-II) to microtubule-associated protein 1A/1B-light chain 1 (LC3-I) ratio and LC3-II/LC3-I AUCi (potentially elevating autophagic activity) when compared to normal-weight subjects in response to maximal aerobic exercise [29], along with a positive relationship BMI, waist/hip ratio, and fasting insulin levels [29]. However, evaluation of autophagic modulators need to be utilized to fully assess autophagic flux [30]. More recently, we demonstrated young adults with obesity had similar responses to both acute traditional continuous moderate intensity exercise and acute high-intensity interval exercise in adipokines (e.g., C1q-TNF related protein 9 [CTRP9]) compared to normal-weight controls, which has anti-inflammatory effects and improves endothelial function [31]. Additionally, we also show that young adults with obesity have an attenuated response to pentraxin 3 (PTX3; an anti-inflammatory mediator released by neutrophils into circulation) compared to normal weight individuals in response to acute high-intensity interval exercise [32]. However, it is known that exercise training can improve overall health in obese older adults by lower body fat and systemic inflammation [33], which is partially supported by our recent results with an improvement of body weight and BMI following 12 weeks of both swimming and cycling exercise training, with no change in appetite stimulating hormones (e.g. leptin) [34]. A meta-analysis demonstrated that increased physical activity improves body composition and insulin-like growth factor 1 (IGF-1) in older adults with sarcopenic obesity [35]. These increased levels of IGF-1 also inhibit astrocyte response to inflammatory insult, thereby modulating neuroinflammation [36]. Exercise training attenuates obesity-associated meta-inflammation [37] and modifies metabolic hormones that may counteract chronic inflammation and obesity-related conditions [38]. With just 16 weeks of aerobic exercise training, CRP was significantly decreased in obese young women [39]. A similar study showed that 10 months of aerobic exercise training significantly decreased CRP, IL-6, and IL-18 in overweight older adults [40]. In older adults with obesity and diabetes, 6 months of aerobic exercise training decreased CRP and IL-18 with an increase in the anti-inflammatory cytokine IL-10 [41]. A recent meta-analysis demonstrated that exercise interventions also attenuate inflammaging (IL-6, CRP, and TNF-α) in middle-aged and older adults with type 2 diabetes [42]. Studies utilizing animal models provide organ-specific mechanistic insight (e.g., liver, lung, stomach) for improvements in inflammation, oxidative damage, cellular senescence, and hepatic function after 3 months of aerobic exercise training via changes in sirtuin activity, nuclear factor kappa B (NF-κB), and peroxisome proliferator-activated receptor-gamma coactivator (PGC-1α) [43]. Collectively, these findings demonstrate that long-term aerobic exercise interventions are an effective method to reduce systemic inflammation in obese older adults.

Moreover, resistance exercise can also be used to combat obesity and age-related chronic inflammation. One year of resistance exercise training has shown to significantly decease CRP levels and increase adiponectin concentrations in young overweight women [44]. In fact, a study with just 10 weeks of resistance training demonstrated a decrease in CRP with overweight and obese individuals [45]. Prior evidence found decreases in CRP and IL-6 after resistance training for 18 months in obese older adults but not with aerobic training [33]. Furthermore, a meta-analysis showed resistance training decreasing CRP and tended to lower IL-6 levels in older adults [46]. As such, overweight older adults with higher levels of physical activity express lower concentrations of IL-6 independent of weight loss [47]. These studies support the utilization of resistance exercise to improve systemic inflammation in older adults with obesity, with the added benefit of attenuating sarcopenia commonly seen with this population.

**Figure 1:**
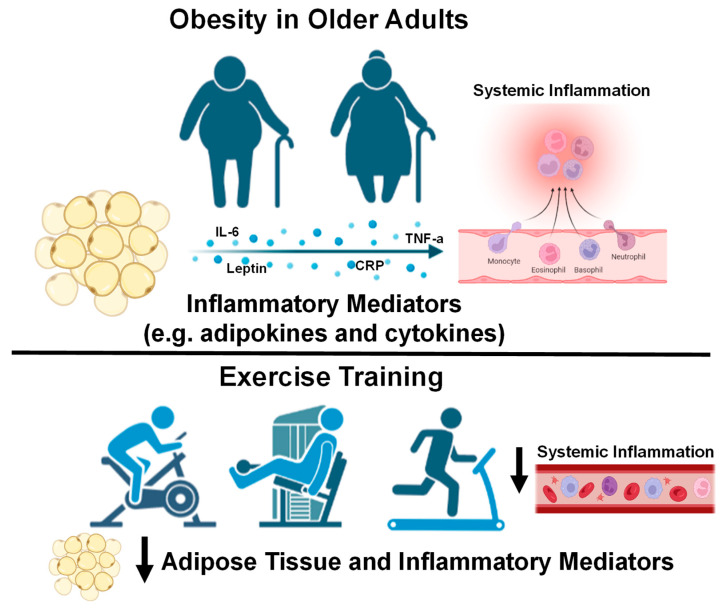
Older adults with obesity and the effect of exercise interventions on systemic inflammation. Down arrows indicate decreases in systemic inflammation with exercise training. Images created with Biorender.com accessed on 13 March 2023.

Indeed, a combination of diet, aerobic, and resistance exercise training is likely to be the most effective lifestyle modification aimed at improving visceral adipose tissue, systemic inflammation, blood pressure (BP), lipid profile, and insulin sensitivity in obese older adults [48]. For example, a 1 year diet and exercise intervention improved insulin sensitivity more than diet or exercise alone in obese older adults [49]. This study also demonstrated decreases in CRP, TNF-α, and increases in adiponectin concentrations after the combined diet and exercise intervention [49]. Reductions in visceral adipose tissue that are hormonally active in obese adults are mediated by IL-6 released from skeletal muscles with exercise, which also acts in an anti-inflammatory manner to reduce chronic systemic inflammation [50]. Another study using combined aerobic and resistance exercise training exhibited reduced immune cells and inflammation (TNF-α and IL-8) in subcutaneous adipose tissue with decreased levels of hypoxia-inducible factor 1-α and superoxide dismutase, a biomarker of oxidative stress, in overweight females [51]. Similarly, a combination of aerobic and resistance exercise training for 12 months in obese diabetic older adults decreased CRP and risk factors for cardiovascular disease (CVD) [52]. Additionally, 15 weeks of caloric restriction and aerobic exercise training significantly reduced systemic inflammation (with decreases in CRP, IL-6, TNF-α, IL-8, monocyte chemoattractant protein-1 [MCP-1]), subcutaneous adipose tissue inflammation, increased adiponectin and insulin sensitivity in severely obese participants [53]. A recent systemic review highlighted improvements in inflammatory biomarkers in adults with obesity using aerobic exercise training (decreased CRP, IL-6, and TNF-α), resistance training (decreased TNF-α), and concurrent training including both aerobic and resistance training (decreased TNF-α) [54]. In overweight and obese middle-aged and older adults, 12 weeks of aerobic training alone, resistance training alone, or in combination decreased TNF-α [55]. Collectively, these studies demonstrate that lifestyle interventions including diet, aerobic and resistance exercise training are very effective at reducing body fat and improving systemic inflammation in older males and females (Figure 1).

## 3. Aging: Cardiovascular Responses to Exercise in Obesity

Excess adipose tissue increases cardiovascular (CV) strain which, over time, places profound stress on the CV system. CV late effects, such as stroke, left ventricular hypertrophy, heart failure (HF), and various forms of cardiomyopathies, are highly prevalent with age-related obesity [56,57,58,59]. In fact, more than 80% of patients with HF with preserved ejection fraction (HFpEF), a cardiac consequence of chronic hypertension, are overweight or obese [60]. There is also a reduction in the elastic properties of the arteries leading to stiffening, termed arterial stiffness, which is associated with abdominal obesity [61] and is independently associated with CVD and mortality [62]. Additionally, obesity-related hypercholesterolemia increases plaque buildup within the myocardial vasculature leading to dysrhythmias, coronary artery disease, myocardial ischemia and infarction, and premature death [63,64]. Properly functioning endothelial cells that line the lumen of arteries, arterioles and capillaries are crucial for controlling systemic blood flow, BP, and prevents the development of atherosclerosis via nitric oxide production, a potent vasodilator, anticoagulant, and antithrombotic biomolecule [65,66,67,68]. Endothelial dysfunction is a modifiable CV risk factor and a hallmark characteristic of accelerated vascular aging that is associated with obesity-related hypertension and CV disease (CVD) [69,70,71]. Endothelial dysfunction is also associated with sarcopenia, the age-related loss in muscle mass, strength and/or function, where there is less nutritive blood flow to skeletal muscles from reduced capillarization [72]. Sarcopenia is highly related to the development of hypertension [73], arterial stiffness [74,75], endothelial dysfunction [76], and CVD via physical inactivity and inflammation [77,78]. Sarcopenic obesity is more catastrophic than sarcopenia and obesity alone [79] and is becoming increasingly prevalent in the sedentary aging population, where there is a large imbalance of adipose-to-muscle tissue ratio and is associated with disability, CVD and mortality [80,81]. Low muscle strength (dynapenia) occurs independently of the loss in muscle mass [82] and is highly associated with poor physical function [83], quality of life [84], mortality [85], and fall risk [86,87] in obese middle-aged and older adults. Older adults diagnosed with CVD have a high prevalence of dynapenia which is associated with increased mortality rate, highlighting the prognostic utility of dynapenia in patients with CVD [88]. Dynapenia is also highly correlated with endothelial dysfunction in elderly women [89], demonstrating early-onset CV consequences related to low muscle strength. Unfortunately, obesity-related CVD prevalence is still on the uprise [90] and is attributed to factors like physical inactivity and diets high in saturated fats and refined carbohydrates [91,92]. Moreover, endothelial dysfunction is the earliest vascular maladaptation with obesity [93], which progressively worsens with age [94]. Similarly, obesity across the lifespan leads to increased arterial stiffness [61,95,96]. However, age- and obesity-associated disorders may be improved with lifestyle interventions that promote weight loss, such as physical activity and exercise. To combat the severe CV consequences of obesity in older adults, the United States Department of Health and Human Services recommends that adults ≥18 years old participate in ≥150 min/week of moderate or ≥75 min/week of vigorous aerobic exercise to reduce all-cause mortality risk [97]. 

Exercise capacity and tolerance in obese adults are less than their lean counterparts. In a cohort of obese women, acute submaximal capacity, peak aerobic capacity and recovery rate were reduced compared to lean controls [98], demonstrating the acute obesity-related reduction in CV responses to physical activity. An explanation for this may be chronotropic incompetence, which can be defined as the inability to increase heart rate to meet blood flow demands of the working muscles during exercise and is an independent predictor of major CV outcomes and mortality in obese adults [99,100,101]. Exaggerated sympathetic nerve activity at rest and during exercise may provide further explanation for the abnormal increases in heart rate (HR) and BP at rest, as well as the delay in returning to basal levels post-exercise [102,103,104,105]. Furthermore, impaired endothelial function has been seen in sedentary obese women after a bout of strenuous weightlifting compared to lean controls [106], suggesting that obesity compromises the functionality of the peripheral vasculature and promotes possible vascular risk when high-intensity exercise is performed. One benefit after acute aerobic [107] and resistance exercise [108] is a sustained reduction in BP for up to 24 h, termed postexercise hypotension, although these reductions in BP are short-lived. Complications hinder obese older adults from exercising for long durations, including discomfort, reduced motivation for exercise, and decreased adherence to exercise training [109,110]. Since chronic exercise is the most effective route to reduce excess adiposity and improve overall CV health, the adverse CV responses to acute exercise are important to monitor as obese adults begin exercise training. 

Repeated bouts of aerobic and resistance exercise, or a combination of both, are crucial to improve exercise tolerance and overall CV health in obese populations. Participating in regular exercise training can enhance functional characteristics of the CV system and begin to reverse CV risk associated with obesity. Chronic exercise training improves vascular function such as endothelial function [111,112], exercise performance [113,114], overall CV health, and modulates cardiac autonomic function in obese adolescents [115,116,117,118]. Existing literature also show the beneficial CV adaptations to exercise training in the adult obese population. Regarding aerobic exercise training, Amano and colleagues conducted a 12-week moderate-intensity aerobic exercise training intervention in middle-aged obese adults and found reductions in body fat, preserved muscle mass, increased aerobic capacity, and improved parasympathetic activity via increased HR variability [119]. These results suggest a “reset” mechanism of the autonomic nervous system (such as improved baroreflex sensitivity) and significant improvements in CV health after only 12 weeks of aerobic exercise training in obese adults. In another intervention-based exercise training study, high-intensity aerobic exercise training elicited the largest reduction in systolic BP during exercise after 6 months versus low and moderate intensities in obese postmenopausal women [120]. Six months of aerobic exercise training also improved endothelial function in hypertensive obese postmenopausal women regardless of intensity [121]. Resistance exercise training alone in obese adults have also yielded improvements in CV health. Figueroa and colleagues tested the effects of a 12-week whole-body vibration training program, a form of resistance exercise, in hypertensive obese postmenopausal women and found decreases in leg arterial stiffness which were associated with reduced ankle and aortic systolic BP [122]. Twelve weeks of low-intensity resistance exercise training also reduced mean arterial pressure in obese postmenopausal women, along with reductions in systemic arterial stiffness when exercise was combined with a hypocaloric diet [123]. These findings are important to consider, since hypertension is a hallmark risk factor of obesity, nearly 40% of postmenopausal women are obese [124], and more than 75% of women ≥60 years are hypertensive [125,126,127]. Although it can be speculated that hypertension may be due to reduced estrogen, hormone replacement therapy shows no effect and possibly an increase in BP [128,129], demonstrating no cardioprotective traits compared to women not on hormone therapy [130,131,132]. These findings suggest that obesity, and not age-related reductions in sex hormones, is associated with hypertension in postmenopausal women. A combination of moderate-intensity aerobic and resistance exercise training for 12 weeks has shown to improve cardiorespiratory fitness in middle-aged obese adults [133]. Park and colleagues also demonstrated that combined aerobic and resistance exercise at moderate intensity for 12 weeks reduced systolic and diastolic BP, mean arterial pressure, pulse pressure, systemic arterial stiffness, low-density lipoprotein cholesterol, and increased aerobic capacity in obese older men [134]. Data from the Maastricht study also found that 12 weeks of combined aerobic and resistance exercise improved oxygen uptake, left ventricular ejection fraction and cardiac lipid profile in overweight/obese middle-aged males [135]. Results from these studies suggest that a combination of aerobic and resistance exercise training for at least 12 weeks may yield more robust improvements in CV function in obese middle-aged and older adults compared to performing aerobic or resistance exercise alone.

Lifestyle changes (diet and exercise training) are suggested to be the most optimal strategy to improve physical limitations [136] and systemic inflammation [137] in sarcopenic/dynapenic obese adults. It is recommended that combined aerobic and resistance exercise training should be individually, progressively prescribed for sarcopenic obese adults to improve muscle mass and function [138]. However, to date, only one study in obese sarcopenic rats has examined the effects of exercise training (aerobic, 20 weeks) on CV health [139]. A study performed by Sénéchal and colleagues found that 12 weeks of a combined hypocaloric diet and resistance exercise training intervention improved body weight (due to reductions in fat mass), waist circumference, blood lipid profile, and systolic BP along with enhanced physical capacity in 38 dynapenic obese postmenopausal women [140]. Although it is known that sarcopenic obesity could contribute to exercise intolerance and reduced cardiorespiratory fitness in HF patients [141,142], identifying the benefits of exercise training (aerobic, resistance, and combined) on CV health in sarcopenic/dynapenic obese adults are of critical need. Mechanistically, oxidative stess seem to play a critical role in exacerbating the negative effects of sarcopenic obesity [143], which is associated with CVD risk [80,144,145]). Resistance exercise training has shown to decrease oxidative damage and stress in older adults [146,147]. Recently, it has been demonstrated that 8 weeks of low-intensity resistance exercise training with slow movement combined with L-citrulline supplementation improved leg lean mass and leg curl strength, and L-citrulline alone improved leg endothelial function in hypertensive postmenopausal women. Although women in this study were not diagnosed with sarcopenia, they were physically inactive [148]. L-citrulline is a key promoter of endogenous nitric oxide production, a potent antioxidant [149], provides vascular and muscular benefits to exercise training in older adults [150], and is suggested to be combined with exercise to manage sarcopenia [151]. Given these findings, L-citrulline supplementation paired with aerobic, resistance or combined exercise training may yield improvements in muscle mass, function, and CV health in sarcopenic and/or dynapenic obese adults.

**Figure 2:**
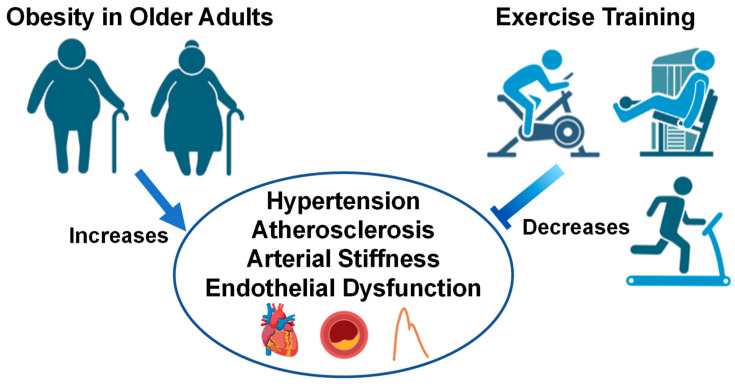
Older adults with obesity and the effect of exercise interventions on cardiovascular health. Images created with Biorender.com accessed on 13 March 2023.

Epidemiological data show marked reductions in 10-year CVD risk in overweight and obese middle-aged adults who perform ≥150 min/week of moderate-to-vigorous exercise [152]. This is in line with the US Physical Activity Guidelines, which established that ≥150 min/week of moderate- or ≥75 min/week of vigorous-intensity aerobic physical activity reduces CVD morbidity and mortality, BP and incidence of hypertension, weight loss and prevention of weight loss, type 2 diabetes prevalence, and an adverse blood lipid profile [153]. When examining obesity-related CVD, evidence from the Cochrane database show reductions in CVD-related mortality and decreased risk of myocardial infarction from exercise-based cardiac rehabilitation programs ranging from 6 months to >3 years [154]. Further, 6 months to 1 year of community-based cardiac rehabilitation has shown to improve left ventricular function, exercise time, and mobility in patients who experienced prior acute myocardial infarction [155]. Further, 20 weeks of aerobic exercise training has shown to improve peak aerobic capacity in obese older patients with HFpEF [156]. These results are important to consider, since obesity is associated with blunted skeletal muscle blood flow during low-intensity exercise in middle-aged and older HFpEF patients, which may contribute to exercise intolerance in this population [157]. Since a high percentage of HFpEF patients are obese [60], exercise training is suggested to improve exercise tolerance in this patient population, and lower risk of all-cause mortality in this patient population [158], but more research is needed to elucidate the CV mechanisms behind the beneficial effects seen in the literature thus far. Altogether, these studies show substantial improvements in exercise tolerance, aerobic capacity, CV function and, importantly, reductions in CVD and risk factors in obese adults after long-term exercise training as illustrated in Figure 2. Evidence from this section suggests that although acute exercise unveils physiological abnormalities, chronic exercise training, preferably combined aerobic and resistance, can improve obesity-related CVD in young and old obese populations. 

## 4. Neurobiological Responses in Older Adults with Obesity and Effects of Exercise

Within the central nervous system, eating behavior is thought to be largely regulated by an intricate interaction between the circuitry that regulates energy intake requirement and reward pathways [159]. In this context, the hypothalamus is seen as a key hub in the regulation of hunger and satiety cues [160], while the mesolimbic and mesocortical dopaminergic pathways (highly activated by palatable food) are crucial for the positive behavioral response to food consumption (e.g., food-associated reward) [159,161]. Found in the forebrain, the hypothalamus is one of the smallest structures in the human brain, integral for key physiological processes such as thermoregulation, neuroendocrine integration, energy metabolism, and energy expenditure [162]. Traditionally, the hypothalamus has been functionally divided into thirds, and components of the tuberal hypothalamus are credited with the regulation of hunger and satiety [159,162]. Since the tuberal hypothalamus contains the arcuate nucleus, the venteromedial, and the lateral areas, it contains neurons that control hunger and food intake including proopiomelanocortin (POMC) and agouti-related peptide and neuropeptide Y (AgRP/NPY) neurons [159,160]. POMC neurons are stimulated by the secretion of peripheral hormones such as adipocyte-derived leptin and pancreatic-derived insulin, and function to regulate satiety following food consumption [163]. On the other hand, as their name implies, AgRP/NPY neurons are associated with the generation of orexigenic peptides AgRP and NPY that stimulate appetite during states of energy deficits such as fasting [164,165]. Importantly, inhibition of POMC neurons, via knockout or ablation, leads to uncontrolled food consumption and weight gain [166,167], while ablation of AgRP/NPY neurons leads to anorexia and starvation in mice [168]. From an aging standpoint, aged rats were previously reported to have significantly lower POMC neuronal activity within the hypothalamus and higher weight gain [169] while increasing POMC tone in aged rats was associated with weight loss, lower visceral adipose deposits, and improved fat metabolism [170]. Intriguingly, aged rats have also been reported to have lower protein expression of NPY protein in the hypothalamus [171] and reduced AgRP gene expression in response to fasting [172]. However, it is important to note that although fasting can lower hypothalamic gene expression of AgRP in old rats, intracerebroventricular injection of AgRP in old rats led to similar levels of food intake as those seen in younger rats exposed to intracerebroventricular injection of AgRP [172]. As such, chronic obesity may first lead to early adaptations that hinder the neurobiological mechanisms behind food satiety that, along with the process of biological aging, may lead to accrued energetic imbalances that favor the development of sarcopenic obesity and obesity-related metabolic syndromes [169]. Although not fully understood, research has found that obese individuals have elevated circulating insulin and leptin while decreased circulating ghrelin [160]. These changes in circulating hormones may, in turn, lead to insulin and leptin resistance within the tubular hypothalamus causing reduced POMC neuronal activity and undisrupted AgRP/NPY neuronal activity, possibly resulting in sustained hunger [160,165]. This is substantiated by data showing that obese older adults display hypothalamic insulin and leptin resistance [173,174], which can, in turn, facilitate hypothalamic neuroinflammation and neuronal stress [175]. In fact, it is well established that microglial cells express leptin receptors, and leptin resistance can induce the release of inflammatory mediators such as NF-kB, IL-1β, and TNF-α by microglial cells [176]. Moreover, because plasma leptin has been shown to positively associate with the number of activated microglia in the hypothalamus of mice fed with a high fat diet, it is possible that in obesity, insulin resistance can lead to not only dysregulated peripheral immune responses, but also maladaptive neuroinflammatory states that ultimately contribute to energy imbalance and diet-induced obesity [177]. However, it must be kept in mind that that advanced chemogenetic and optogenetic approaches suggest the roles of AgRP/NPY and POMC neurons are not fully antagonistic, and their activity and behavioral consequences (eating behaviors) may be modulated by stimuli such as sustained environmental stress [178] and thus, other external stimuli may also lead to neurobiological adaptations in obesity that disrupt the neuroendocrine and neuroimmune regulation of food intake. Importantly, it must also be noted here that hypothalamic POMC and AgRP/NPY circuits may also fluctuate during biological aging and facilitate imbalances in energy metabolism [169]. Indeed, hypothalamic POMC neuronal activity is significantly lower in aged rodents when compared to younger counterparts [169] and AgRP/NPY receptor levels and signaling have been observed to fluctuate in aged rats based on feeding status [179]. Unsurprisingly, imbalances in energy metabolism are commonly found during biological aging, which can, in turn, lead to changes in body composition and give rise to conditions such as sarcopenic obesity [2]. Defined, sarcopenic obesity is an age-related chronic condition characterized by decreases in skeletal muscle mass and function concomitant to increases in high body fat and loss of appetite [2]. Although not exclusively, the age-related neurobiological changes in POMC and AgRP/NPY hypothalamic circuitry have been postulated to partially contribute to the energy imbalance and loss of appetite seen during sarcopenic obesity [2]. However, to which degree remains poorly understood, and should be studied further. Lastly, emerging lines of evidence suggest there may be differences in the neurobiological adaptations during obesity based on biological sex [180]. For example, recent work by Freire-Regatillo and colleagues found that POMC and AgRP/NPY adaptations to high fat diet differed based on sex in middle-aged TgAPP mice [180]. Specifically, following a high fat diet, female mice had significantly greater increases in body weight, visceral and subcutaneous adipose tissue, as well as higher expression of POMC mRNA when compared to males, and males displayed lower AgRP/NPY mRNA than females [180]. As such, the neurobiological systems that regulate energy homeostasis and appetitive behaviors are not only dysregulated during obesity but can also be influenced by biological aging, sex, and even environmental stress. Thus, future research should strive to better characterize how these neurobiological systems are influenced from an integrative point of view.

Meanwhile, the mesolimbic and mesocortical pathways are both dopaminergic components of the brain’s reward circuitry that are heavily involved in addictive behavior such as substance abuse [181,182]. In fact, because these two pathways are activated by highly palatable foods and have been implicated in behavioral overeating [159,183], the concept of food addiction continues to be heavily scrutinized as another possible neurobiological adaptation in chronic obesity [184]. Although relatively novel and somewhat controversial, food addiction is an important concept to recognize given that obese individuals display behavioral constructs associated with addiction such as impulsivity, increased sensitivity to reward, and heightened need to consume for pleasure (e.g., food cravings and hedonic eating) [185,186]. From a neuroanatomical point of view, the homeostatic circuitry of food intake (hypothalamus) and the reward pathways display a large degree of interconnectivity [159] and data from animal studies indicate structures within the mesolimbic pathway such as the nucleus accumbens can lead to increased appetitive behavior [187]. Thus, it is possible that the brain’s reward circuitry may influence food intake, give rise to compulsive food consumption, and increase the risk of developing obesity [186,188]. This notion is supported by human and animal studies showing dopaminergic release becoming altered in the presence of food cues and is associated with the motivation of wanting to seek food consumption [189,190,191]. There is also data to suggest that obesity leads maladaptive changes in reward circuitry similar to those found in substance abuse, such as decreases in dopamine receptors, that may trigger compulsive food intake [192]. Moreover, human studies have found that when compared to lean counterparts, obese individuals display lower activation of reward circuitry during food consumption, higher somatosensory activation in the presence of food cues, and greater anticipation of food consumption [193]. Together, these lines of evidence showcase similarities with models of substance abuse whereby a mismatch in the expected reward of food consumption leads to compulsive eating in an attempt to seek reward [194], possibly explaining why food addiction is gaining traction as a likely etiological component of obesity. 

**Figure 3:**
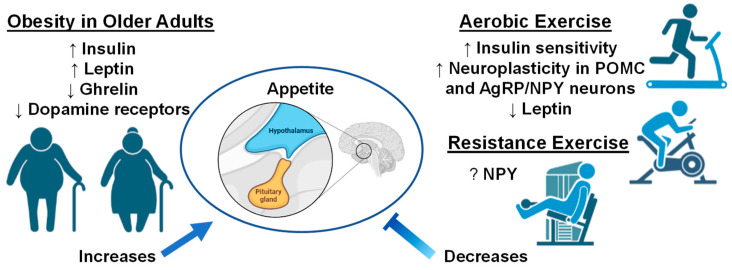
Older adults with obesity and the effect of exercise interventions on appetite. Up arrows indicate increases, down arrows indicate decreases, and question mark indicates unknown if changes occur with exercise interventions. Images created with Biorender.com accessed on 13 March 2023.

The benefits of aerobic exercise towards brain health and synaptic plasticity are well established in the literature [195,196]. Unsurprisingly, exercise is seen as a powerful tool to combat the prevalence of obesity [195] given that in obese older adults, chronic exercise training can improve insulin sensitivity, reduce inflammation, and improve body composition [197,198]. Indeed, chronic exercise training is postulated to improve energy balance through modulation of neuroplasticity in POMC and AgRP/NPY neurons [199,200]. A recent study in mice provides compelling evidence showing aerobic exercise can increase POMC neuronal excitability, with leptin-dense POMC neurons showing the most robust response to exercise [201]. Intriguingly, in this same study, exercise was reported to inhibit NPY neuronal activity, suggesting that exercise can exert rapid reorganization in multiple neuronal populations within the tuberal hypothalamus [201]. Further, there is limited evidence to suggest POMC mRNA expression is differentially altered following acute and chronic aerobic exercise in not only the hypothalamus, but also the frontal cortex and hippocampus [200]. Together, these lines of evidence suggest aerobic exercise can facilitate neuroplasticity within POMC neurons throughout the brain, and ultimately benefit the efficacy of satiety cues. Moreover, although not specific to the hypothalamus, human data also suggests exercise-related reductions in leptin and high-density lipoprotein, as well as increases in serum brain derived neurotrophic factor (BDNF) which are all related to improved gray and white matter integrity within the brain, suggesting improved exercise-modulated neuroplasticity in obese humans [202]. Moreover, it is also worth noting several independent research groups have found, in animal models, that aerobic exercise can be neuroprotective to obesity-related neuroinflammation within the hypothalamus [203,204], which may ultimately further facilitate the reestablishment of satiety signaling within the brain [205]. Intriguingly, there is evidence to suggest the effects of exercise may differ between sexes. In a recent study, Wilson et. al., (2020) found that in obese mice, only female mice downregulate mRNA expression NPY within the hypothalamus following 12 weeks of aerobic exercise and intermittent fasting [206]. However, it is worth noting that the ways by which exercise may differentially influence POMC and AgRP/NPY systems based on sex is a relatively novel concept that needs to be studied extensively. Lastly, there is limited data to suggest that in obese adults, chronic resistance exercise training may improve circulating biomarkers associated with appetitive behavior such as glucose and NPY, as well as reduce circulating pro-inflammatory markers such as TNF-α and IFN-γ [207,208]. Moreover, while randomized controlled exercise trials in humans are limited in individuals with sarcopenic obesity, a combination of aerobic exercise and resistance training may be the most beneficial [209]. For example, Villareal and colleagues found that in dieting obese older adults at risk of developing sarcopenia, performance in the physical performance test improved more robustly in older adults that performed concurrent exercise training than in aerobic or resistance training alone [210]. Meanwhile, in a smaller study including individuals diagnosed with sarcopenic obesity, concurrent exercise training was found to induce similar improvements in body weight and fat mass, while resistance training alone yielded greater improvements in muscular strength than aerobic or concurrent training [211]. Together, these studies suggest that exercise training routines that incorporate resistance training or a combination of resistance training plus aerobic exercise will improve the clinical outcomes in obese older adults and adults with sarcopenic obesity. However, it is important to highlight here that whether resistance exercise can more directly benefit the neurocircuitry affected in obesity remains understudied in the literature. Indeed, future studies should aim to bridge this knowledge gap from an acute and chronic point of view with an emphasis on neuro-centric biomarkers to better understand whether resistance exercise can serve as an ancillary tool against obesity. Nevertheless, although not fully elucidated, it is possible that exercise may aid in re-establishing a balance in the brain circuitry that regulates hunger and food intake as illustrated in Figure 3. There is ample evidence demonstrating that aerobic and resistance exercise training can be beneficial in older adults, adults with obesity, and older adults with sarcopenic obesity. However, it remains challenging to address in humans the neurobiological mechanisms by which exercise can influence the neurocircuitry behind energy homeostasis and appetitive behavior. Nevertheless, animal studies continue to be crucial in bridging this gap in the literature and provide compelling evidence that exercise can act directly on POMC and AgRP/NPY neurons, as well as by influencing neuroinflammatory mediators such as pro-inflammatory cytokines. Moreover, emerging frameworks suggest there may be sex differences in some of the mechanisms discussed above, yet to what extent remains to be fully elucidated. Future studies would benefit from addressing some of the gaps in the literature highlighted in this section to better characterize the neurobiological responses to obesity, sarcopenic obesity, possible sex differences, and how exercise influences each.

## 5. Conclusions

Obesity and aging are involved in a decline in cognitive function underlying the inflammatory mechanisms; however, the literature regarding how both interact and impact this complex process of cognitive impartment across the lifespan still remains elucidated. Although obesity is a reversible disorder through lifestyle changes, it is important to note that early interventions are crucial to prevent accelerated CV aging and reduce the risk of severe CV late effects seen in the aging obese population. Lifestyle modifications such as physical activity should be considered as a main intervention to minimize the synergistic effect of obesity on age-related conditions as summarized in Table 1, such as cerebrovascular disease. Of particular note, obesity has been recently shown to be a primary predictor for a long-term risk of cerebrovascular mortality in black vs. white individuals [212]. Thus, future investigation should also explicate the influence of racial/ethnic disparities in the development of related diseases to better understand how obesity accelerates biological changes in brain health across the life course. 

## Figures and Tables

**Table 1:** Exercise Interventions in Obese Middle-Aged and Older Adults.

Inflammatory Responses			
Reference	Population	Type/Duration of Study	Conclusions
Balducci et al., 2012 [52]	Overweight and obese diabetic middle-aged and older adults (N = 36)	Aerobic and resistance exercise (75 min of aerobic exercise and four resistance exercises) for 12 months	Decreased CRP, hemoglobin A(1c), BMI, waist circumference, blood pressure, LDL cholesterol, and the coronary heart disease risk score
Bruun et al., 2006 [53]	Severely obese adults (N = 27)	Aerobic exercise (2–3 h, 5×/week) for 15 weeks	Decreased CRP, IL-6, TNF-α, IL-8, MCP-1, and body weight Increased insulin sensitivity, and adiponectin
Čížková et al., 2020 [51]	Overweight older females (N = 51)	Aerobic and resistive training (3×/week) for 4 months	Decreased IL-8, TNF-α and adipose tissue Increased insulin sensitivity
Donges et al., 2010 [45]	Overweight and obese adults (N = 76)	Aerobic training (N = 41) or resistance training (N = 35) for 10 weeks	Decreased CRP with resistance training and a trend for reduced CRP with aerobic training
Ho et al., 2013 [55]	Overweight and obese middle-aged and older adults (N = 48)	Aerobic (N = 15), resistance (N = 16), or combination exercise (N = 17) for 12 weeks	Decreased TNF-α with all exercise interventions
Kadoglou et al., 2007 [41]	Overweight and obese diabetic older adults (N = 30)	Aerobic exercise (45–60 min, 4×/week) for 6 months	Decreased CRP and IL-18 Increased IL-10 No change in TNF-α and adiponectin
Kohut et al., 2006 [40]	Obese older adults (N = 48)	Aerobic exercise (45 min, 3×/week) for 10 months	Decreased CRP, IL-6, IL-18 and TNF-α
Marcell et al., 2005 [213]	Overweight and obese insulin resistant middle-aged adults (N = 37)	Aerobic exercise (30 min, 5×/week) for 16 weeks	No change in CRP Increased adiponectin
Rejeski et al., 2019 [33]	Overweight and obese older adults (N = 154)	Aerobic training (N = 79) or resistance training (N = 75) for 18 months	Decreased CRP and IL-6 with resistance training
**Cardiovascular Responses**			
**Reference**	**Population**	**Type/Duration of Study**	**Conclusions**
Amano et al., 2001 [119]	Obese middle-aged males and females (N = 18)	Aerobic exercise (30 min/session, 3×/week) for 12 weeks	Decrease in BMI, % fat Increase in aerobic capacity, sympathetic, and parasympathetic nerve activity
Figueroa et al., 2015 [122]	Obese postmenopausal women with high blood pressure (N = 36)	Whole-body vibration training stratified by ankle systolic blood pressure into 3 groups (high blood pressure, normal blood pressure, non-exercising control) for 12 weeks	Whole-body vibration training reduced ankle systolic blood pressure in the high ankle systolic blood pressure group Whole-body vibration training improved brachial and aortic systolic blood pressure, leg artery stiffness and whole-body artery stiffness independently of ankle systolic blood pressure vs controls
Figueroa et al., 2013 [123]	Obese postmenopausal women (N = 41)	Low-intensity resistance exercise training, hypocaloric diet, or combined exercise + diet for 12 weeks	Hypocaloric diet reduced whole-body artery stiffness, mainly by decreasing leg artery stiffness, which was related to the loss of trunk fat Exercise + diet improved whole-body artery stiffness and muscle strength and prevented loss of lean body mass compared to exercise alone
Ho et al., 2013 [55]	Overweight/obese middle-aged males and females (N = 64, all participants)	Aerobic exercise for 30 min (group 1), resistance exercise for 30 min (group 2), combined aerobic and resistance (15 min each modality, group 3), and non-exercising controls for 12 weeks	Combined group improved body weight and total body fat compared to resistance exercise and control groups. Combined group also experienced improvements in body fat %, trunk fat %, and aerobic capacity compared to controls
Swift et al., 2012 [121]	Obese postmenopausal women with elevated blood pressure (N = 155)	Aerobic exercise at different energy expenditure doses (4, 8, and 12 kcal/kg/week vs controls) for 6 months	Similar improvements in endothelial function in all exercise groups vs controls Participants with endothelial dysfunction at baseline has significant improvements after intervention compared to those with normal endothelial function
Park et al., 2020 [134]	Obese older males (N = 20)	Combined elastic band resistance and aerobic exercise training 3 days/week vs non-exercising controls for 12 weeks	Exercise group improved body weight, BMI, and body fat % Exercise group also experienced reductions in systolic blood pressure, mean arterial pressure, pulse pressure, and whole-body artery stiffness Plasma low-density lipoprotein, peak aerobic capacity and grip strength were also improved in the exercise group
Schrauwen-Hinderling et al., 2010 [135]	Overweight/obese middle-aged males (N = 14)	Combined aerobic and resistance exercise training for 12 weeks	Left ventricular ejection fraction, plasma glucose, and cardiac lipid content of the septum were all improved after intervention.
**Neurobiological Responses**			
**Reference**	**Population**	**Type/Duration of Study**	**Conclusions**
Chen et al., 2017 [211]	Sarcopenic obese older adults (N = 60) randomized into a control, aerobic, resistance, or concurrent training group.	8-week intervention study. Aerobic training group: 60 min of moderately intense aerobic exercise 2×/week. Resistance training group: series of 10 movements targeting multiple muscle groups at 60–70% 1-repetition maximum, 2×/week.	Skeletal muscle mass, body fat, and visceral fat improved in all exercise groups when compared to controls. Muscle strength improvements greater in the resistance training group. Serum IGF-1 concentration higher in the resistance training group when compared to the control. Concurrent training group displayed greater serum IGF-1 levels than aerobic training or control group.
Gaspar et al., 2018 [204]	6-week-old Swiss mice split into control and obese groups (high fat diet).	Acute treadmill exercise consisting of three 45 min bouts with 15 min. rest between bouts.	In obese mice, serum levels of adiponectin and hypothalamic APPL1 content increased following exercise, while food intake decreased. Exercised Obese mice displayed increased protein expression of molecular mediators such as Akt and TRB3.
He et al., 2018 [201]	Transgenic POMC/NPY mice split into either a sedentary or exercise group	Exercise group intervention: combination of high intensity interval training and continuous exercise. Control group: mice placed on static treadmill for the same amount of time as exercise mice.	Exercise improved depolarization and firing rate of arcuate POMC neurons as well as excitatory inputs to arcuate POMC neurons. Leptin-expressing POMC neurons found to be more responsive to exercise. Exercise found to inhibit NPY neurons. The effects of exercise observed to be transient in NPY neurons but sustainable in POMC neurons.
Jiaxu et al., 2000 [200]	Male Sprague-Dawley rats separated into a sedentary control, and exercise group.	Exercise group: 35 min of treadmill running for 2 weeks after 5-week exercise week acclimation protocol. Control group: no exercise or treadmill placement during entirety of study.	After exercise acclimation period, POMC mRNA expression decreased in frontal cortex and hippocampus following acute exercise bout, but in the hippocampus, mRNA upregulated above baseline levels 30 min post-exercise. Hypothalamic POMC mRNA levels increased immediately after acute exercise bout and remained elevated for up to 3-h post-exercise.
Mueller et al., 2015 [202]	Young overweight/obese women (N = 9)	60 min of concurrent exercise training 2/week for 12 weeks.	Improved circulating metabolic profile post-intervention. Increased levels of circulating BDNF post-intervention. Exercise-dependent changes in metabolic and BDNF levels found to be related to gray matter density in the hippocampus, insular cortex, and left cerebellar lobule. Exercise improved measures of white matter structure.
Onambélé-Pearson et al., 2010 [207]	Sedentary older adults (N = 30)	Participants split into either a low or high resistance exercise intervention 3×/week for 12 weeks.	Improvements in physical function in both groups. Serum levels of NPY found to significantly decreased post-intervention in both groups. Serum levels of IGFBP-3 and TNF-α found to significantly decrease post-intervention in the low resistance group only.
Roh et al., 2020 [208]	Obese older women (N = 26)	Resistance training 3×/week for 12 weeks, or no resistance exercise control.	Improved body composition in resistance training group post-intervention. Lower levels of pro-inflammatory markers (TNF-α and IFN-γ) post-intervention in resistance training group. Higher levels of circulating BDNF and VEGF found post-intervention in resistance training group.
Villareal et al., 2017 [210]	Obese older adults (N = 141) randomized into a control, aerobic, resistance, or concurrent training group, the latter three with added weight management programs.	26-week intervention study. Control group: educational sessions about diet. Aerobic group: weight management program + 60 min of aerobic exercise 3×/week. Resistance group: weight management program + 40 min of resistance exercise 3×/week. Concurrent training group: weight management program + aerobic and resistance exercise training 3×/week.	Physical performance test improvements higher in the concurrent training group than in the aerobic or resistance training groups. Fitness (Peak oxygen consumption) higher in concurrent and aerobic training groups. Muscular strength increased in concurrent and resistance training groups.
Wilson et al., 2020 [206]	Male (N = 39) and Female (N = 49) C57BL/6 mice with high fat diet assigned to 12–24 weeks of Intermittent fasting, high intensity interval training, intermittent fasting + high intensity interval training, or control group.	Intermittent fasting group: 2 alternative days fasting/8-day cycle. High intensity interval training group: 3 days of 6–8 repetitions 20 s. sprints, 40 s. walking/ 8-day cycle. Intermittent fasting + high intensity interval training group: 2 non-consecutive days fasting/ 8-day cycle + high intensity interval protocol 3 non-fast days/ 8-day cycle.	Both intermittent fasting, high intensity interval training, and intermittent fasting + high intensity interval training resulted in significantly less weight gain, adipose tissue accumulation, and lower LDL serum levels. Hypothalamic NPY mRNA expression lower in exercised + fasted females only. Fat oxidation greater in exercised + fasted males, while markers of adipocyte differentiation only decreased in exercised + fasted females.
Yi et al., 2012 [203]	Low-density lipoprotein deficient mice split into exercise (N = 10) or sedentary (N = 6) groups.	Exercise group intervention: 30 min of treadmill running at 10% grade for 26 weeks.	Decreased inflammatory profile found in chronically exercised mice. Lower microglial activation and improved glucose tolerance in exercise mice.

## Data Availability

Not applicable.
